# Nox1/PAK1 is required for angiotensin II-induced vascular inflammation and abdominal aortic aneurysm formation

**DOI:** 10.1016/j.redox.2024.103477

**Published:** 2024-12-19

**Authors:** Hui He, Tianyu Jiang, Meng Ding, Yuan Zhu, Xiaoting Xu, Yashuang Huang, Wenfeng Yu, Hailong Ou

**Affiliations:** Department of Biochemistry and Molecular Biology, School of Basic Medicine, Guizhou Medical University, Gui'an, 561113, Guizhou, PR China

**Keywords:** NADPH oxidase 1, Abdominal aortic aneurysm, PAK1, Smooth muscle cell, Vascular inflammation, MMP2

## Abstract

NADPH oxidase 1 (Nox1) is a major isoform of Nox in vascular smooth muscle cells (VSMCs). VSMC activation and extracellular matrix (ECM) remodelling induce abdominal aortic aneurysm (AAA). In this study, we aim to determine the role of Nox1 in the progression of AAA and explore the underling mechanism. ApoE^−/−^Nox1^SMCko^ mice in which the Nox1 gene was smooth muscle cell (SMC)-specifically deleted in ApoE^−/−^ background, were infused with angiotensin II (Ang II) for 28 days. We found the Nox1 deficiency reduced AAA formation and increased survival compared with ApoE^−/−^Nox1^y/flox^ mice. Abdominal aortic ROS and monocyts/macrophages were reduced in the ApoE^−/−^Nox1^SMCko^ mice after Ang II-infusion. The SMC-specific Nox1 deletion caused less elastin fragments and lower matrix metalloproteinase (MMP) activities in the abdominal aorta. Further, we found the Nox1 protein interacted with p21-activated kinase 1 (PAK1) in Ang II-stimulated VSMCs. The PAK1, controlled by Nox1/ROS, promoted VSMC proliferation, migration and differentiation; this is associated with increased activities of vimentin and cofilin, and cytoskeleton modulation. Moreover, we found that the Nox1/PAK1 activated the downstream MAPKs (ERK1/2, p38 and JNKs) and NF-κB, and upregulated Sp1-mediated MMP2 expression upon Ang II-stimulation. Finally, overexpression of PAK1 in the ApoE^−/−^Nox1^SMCko^ mice increased vascular elastic fibre degradation, pro-inflammatory cytokine expression and AAA incidence. Therefore, we conclude that Nox1, together with PAK1, facilitates Ang II-induced VSMC activation, vascular inflammation and ECM remodelling, and thus potentiates the AAA formation.

## Highlights

• SMC-specific deletion of Nox1 reduces abdominal aortic aneurysm formation.
• Nox1 promotes vascular inflammation and extracellular matrix remodelling.
• Nox1 interacts with PAK1in AngII-induced SMCs.
• Nox1/PAK1is required for SMC activation and inflammation.
• Nox1/PAK1 increases NFκB activation and Sp1-mediated MMP2 expression.

## Graphical abstract

**Figure:**
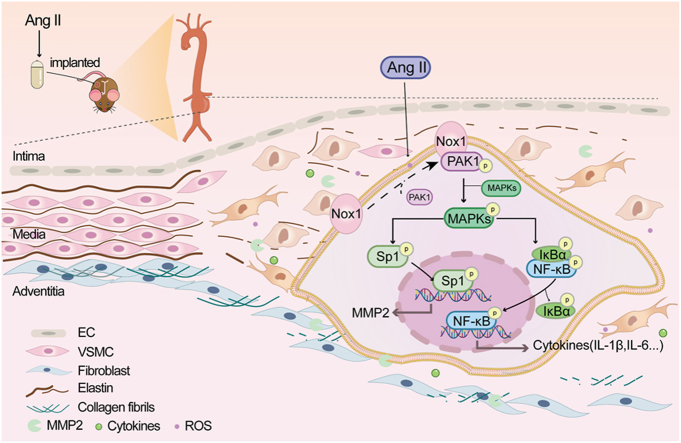

## 1 Introduction

Abdominal aortic aneurysm (AAA) is a permanent and segmental dilation of the abdominal aorta with destructive remodelling of the aortic wall. The main pathological characteristics of AAA include the degradation of elastin and the loss of aortic smooth muscle cell (SMC) functions [1]. Vascular smooth muscle cells (VSMCs) in the media layer are the main cellular components of arteries; these cells provide structural integrity and are crucial for maintaining vessel homeostasis. VSMCs are plastic and undergo phenotypic switching from the quiescent, contractile phenotype to a dedifferentiated, synthetic phenotype in response to injury and various stimuli, in which the cells decreasingly express SMC markers, such as α-smooth muscle actin (α-SMA) and smooth muscle protein 22α (SM22α), with upregulation of genes involved in VSMC activation [2]. Phenotypic modulation is the first step in AAA formation, and subsequent cell proliferation and migration further accelerate the development and progression of various cardiovascular disorders, such as AAA, atherosclerosis and hypertension [3].

Chronic vascular inflammation, monocyte/macrophage infiltration into the endothelium and oxidative stress contribute to AAA formation. Dysregulation of reactive oxygen species (ROS) generation and proinflammatory cytokines such as interleukin-6 (IL-6) and monocyte chemoattractant protein-1 (MCP-1) trigger and promote VSMC proliferation, migration and differentiation [4,5]. Moreover, ROS and cytokines induce the secretion of extracellular matrix (ECM) proteases, including matrix metalloproteinase 2 (MMP2) and MMP9, which increase the proteolysis of ECM components, thus causing vascular abnormalities [6,7].

p21-activated kinases (PAKs) belong to the family of serine/threonine protein kinases, and consists of two groups of six family members: group I (PAK1-3) and group II (PAK4-6) in mammals [8,9]. PAK1 is the major isoform expressed in VSMCs and is activated by the small Rho GTPase family Ras-related C3 botulinum toxin substrate 1 (RAC1) and cell division control protein 42 homologue (Cdc42) [[8], [9], [10]]. Upon stimulation, GTP-bound Cdc42 and Rac1 induce PAK1 autophosphorylation at Thr423 and change the conformation from *trans*-inhibited homodimers to an active, monomeric form [11,12]. Previous studies have demonstrated that stimulators such as angiotensin II (AngII), platelet-derived growth factor BB (PDGF-BB) and thrombin activate PAK1 and trigger signalling cascades regulating cytoskeletal remodelling, migration, and proliferation, which promote vascular remodelling and vessel destabilization [10,[13], [14], [15]].

NADPH oxidase is a main producer of ROS, and seven isoforms (Nox1-5, Duox 1 and Duox 2) have been identified in mammals [16]. Nox1 is highly expressed in the vascular wall and modulates VSMC proliferation, migration and differentiation [[17], [18], [19], [20]]. Abnormal expression of Nox1 is responsible for the development of various vascular diseases, such as hypertension, atherosclerosis and aortic dissection [[21], [22], [23], [24], [25]]. Recently, Nox isoform 1, 2 and 4 were shown to induce eNOS uncoupling and increase AAA expansion [26]. However, the detailed mechanism underlying the effects of Nox1 on AAA formation is not fully understood. In this study, we specifically disrupted Nox1 in VSMCs from ApoE^−/−^ mice and investigated VSMC activation, aortic inflammation and ECM remodelling. We also explored the role of the signalling molecule PAK1 in Nox1-modulated vascular remodelling.

## 2 Materials and methods

### 2.1 Animals

Apolipoprotein E knockout mice (ApoE^−/−^) and Nox1-floxed mice (Nox1^flox/flox^) were obtained from GemPharmatech Company (Nanjing, China). Mice expressing Cre recombinase, which is driven by the smooth muscle 22α promoter (Tagln-Cre mice), were obtained from Cyagen (Suzhou, Jiangsu). Male Nox1 mice with smooth muscle cell-specific deletion (ApoE^−/−^Nox1^SMCko^) were generated by crossing Nox1^flox/flox^ mice with Tagln-Cre mice and ApoE^−/−^ mice. ApoE^−/−^Nox1^y/flox^ littermates were used as controls. After treatments, the mice were anaesthetized and sacrificed by using ketamine (100 mg/kg) and xylazine (10 mg/kg) (I.P.). The experiments were conducted according to the protocols approved by the Ethical Committee for Animal Care and Use of Guizhou Medical University (Approval No. 2200030).

### 2.2 Ang II-induced AAA model

Eight-week-old male mice fed a high-fat diet (HFD, Xietong Bio., Nanjing, China) containing 21 % fat and 0.15 % cholesterol were anaesthetized with chloral hydrate (300 mg/kg) (I.P.), and infused with a 1.44 mg/kg/day dose of Ang II for 28 days via subcutaneous implantation of osmotic minipumps (Alzet Model 2004, Durect Corporation, Cupertino, CA). Sham animals were infused with 0.9 % NaCl solution. At the end of the experiment, the mice were euthanized. The entire aorta was exposed, and the periadventitial tissue was carefully removed under a dissection stereomicroscope (Yongxin, Nanjing, China). The maximum width of the abdominal aorta was measured by using ImageJ after being digitally photographed, and a more than 50 % expansion of the abdominal aorta was defined as an aortic aneurysms [27].

### 2.3 Histological analyses

Abdominal aortic samples were embedded in optimal cutting temperature (OCT) compound and cut into serial cross-sections of 8 μm thickness. The cryosections were then subjected to haematoxylin and eosin (HE) staining. Elastic fibre was stained with Verhoeff–van Gieson (VVG), and the broken elastins were counted. HE and VVG staining was performed as a regular procedure. Images were acquired via microscopy (Nikon, TE2000, Tokyo, Japan), and quantifing by ImageJ software (NIH).

### 2.4 Immunofluorescence and dihydroethidium (DHE) staining

Serial abdominal aortic frozen cross-sections (8 μm) were pretreated in cold acetone for 10 min. After washing with PBS and blocking with 10 % goat serum, the slides were incubated with primary antibodies against Nox1 (1:50, Proteintech, Wuhan, China), α-SMA (1:200, Abcam), CD68 (1:100, Abcam), IL-6 (1:100, Abcam), VCAM-1 (1:100, Proteintech), MMP2 (1:100, Abcam) and *p*-PAK1 (1:100, Abcam) overnight at 4 °C. The slides were washed with PBS three times, and then incubated with a fluorescence-conjugated secondary antibody for 1.5 h at room temperature. Nuclei were counter-stained with DAPI for 5 min. The stained cells were visualized via confocal laser microscopy with wavelengths of excitation and emission at 488/520 nm for α-SMA and IL-6, and 561/570 nm for Nox1,CD68,VCAM-1, MMP2 and *p*-PAK1 (Olympus, SpinSR10, Japan). For DHE staining, the DHE (10 μM, Beyotime, Shanghai, China) was added to the unfixed cryosections, and incubated for 30 min at 37 °C in a dark and humidified chamber. The staining was examined by using fluorescence confocal microscopy with wavelength of excitation at 561 nm and emission at 610 nm. All of the images were analysed by using ImageJ software (NIH, Bethesda, MD).

### 2.5 Zymography analysis

For gelatine zymography, abdominal aortic segments were homogenized and suspended in lysis buffer. Equal-volume samples were mixed with nonreducing loading buffer and run on a 10 % acrylamide-SDS gel containing 0.1 % gelatine at room temperature. The resulting gels were washed with 2.5 % Triton X-100 three times, followed by incubation in developing buffer (50 mM Tris, pH 7.4; 10 mM CaCl_2_; 50 mM NaCl; and 0.05 % Brij 35) for 42 h at 37 °C. The gel was then stained with 0.25 % Coomassie brilliant blue R-250 and photographed. The white bands indicating proteolysis of the gelatine substrate by the MMP were determined via densitometry. For in situ zymography, unfixed aortic cryosections were incubated with 20 μg/mL fluorescein-conjugated gelatine as a substrate (Molecular Probes, Eugene, OR) in developing buffer for 12 h at 37 °C. After washing with PBS and counterstaining the nuclei with DAPI, proteolytic activity or gelatine degradation was detected as green fluorescence (530 nm) via microscopy and quantified by using ImageJ (NIH).

### 2.6 Flow cytometry

Abdominal aortas were digested with lysis buffer containing collagenase, hyaluronidase, and DNase I at 37 °C for 1 h and passed through a 70 μm cell strainer. The single-cell suspensions were incubated with 1 μg of CD16/CD32 (BD Bioscience, San Jose, CA, USA) to block nonspecific binding and stained with 1 μg of antibodies against V500-CD45 (BD), APC-CD11b (BD), PE-F4/80 (BD) and FITC-Ly-6C (BD). The cells were washed with 2 % FCS/PBS and the staining was detected by flow cytometer. Finally, the results were analysed with FlowJo software (BD).

### 2.7 VSMC isolation and culture

The thoracic aortas of 8-week-old wild-type mice were isolated and minced into small pieces. After enzymatic digestion with 1.5 mg/mL collagenase II for 1 h at 37 °C, the dispersed cells were collected and seeded on culture dishes. The cells were maintained in Dulbecco's modified Eagle's medium (DMEM)/F12 (1:1) (Gibco, Grand Island, NY, USA)/20 % fetal bovine serum (FBS, HyClone, Logan, Utah, USA) supplemented with 100 units/mL penicillin and 100 μg/mL streptomycin (Invitrogen, Carlsbad, CA, USA) in a humidified 5 % CO_2_ incubator at 37 °C. Cells at passages 5 to 10 were used in the experiments.

### 2.8 Coimmunoprecipitation and Western blotting

Cells or tissues were harvested and homogenized in RIPA buffer (Beyotime), followed by centrifugation at 12,000×*g* for 10 min at 4 °C. The supernatants were incubated with control or specific antibodies overnight at 4 °C with constant rotation; 20 μl of prewashed protein A/G agarose beads (Beyotime) was then added for an additional 4 h of incubation. After washing, the precipitated proteins were resuspended in 2 × loading buffer and boiled for 5 min to elute them from the beads. The protein complexes were run on SDS‒PAGE, followed by immunoblotting with appropriate antibodies, as indicated. Band intensities were quantified by using NIH ImageJ software.

### 2.9 Pull-down assay

GST-tagged Nox1 and Flag-tagged PAK1 were expressed in BL21 cells and purified by using GST-tag purification resin (Beyotime) and anti-Flag affinity gel (Beyotime), respectively. The purified proteins and the GST resin were mixed together in GST pulldown binding buffer (50 mM Tris-HCl, 200 mM NaCl, 1 mM EDTA, 1 % NP-40, 1 mM DTT, 10 mM MgCl_2_, pH 8.0) for 12 h at 4 °C, followed by PAGE. The gels were stained with Coomassie blue or immunoblotted with antibody against PAK1.

### 2.10 Immunopurification and mass spectrometry

Protein purification was performed via immunoprecipitation by using an anti-Flag affinity gel following the manufacturer's instructions (Beyotime) at 4 °C overnight, and the proteins were eluted with TBS containing 3 × Flag peptide. The precipitates were subjected to liquid chromatography tandem mass spectrometry (LC‒MS/MS) analysis; moreover, some of the proteins were resolved on SDS‒PAGE, followed by silver staining.

### 2.11 Luciferase reporter assay

VSMCs were co-transfected with the MMP2 promoter-driven firefly luciferase construct (pGL3-MMP2) and Renilla luciferase expressing pRL-TK (Promega, Madison, WI, USA). Luciferase activities were measured by using a Dual Luciferase Reporter Gene Assay Kit (Beyotime) on a luminometer (Bio-Tek Instruments, Inc., Winooski, VA, USA) according to the manufacturer's protocol. The blank pGL3 vector was used as control. Each experiment was performed in triplicate and repeated at least three times.

### 2.12 Electrophoretic mobility shift assay (EMSA)

Nuclear extracts were prepared from VSMCs subjected to the indicated treatments and incubated with a biotin-labelled probe targeting the MMP2 promoter (5′-ggggaggagaggggcgggtcggacct-3′) in 5 × EMSA/Gel-Shift binding buffer supplemented with poly (dI-dC) (Beyotime) for 20 min at room temperature. Two hundred-fold molar excesses of unlabelled or mutant probe were used for competitive binding; moreover, 1 μg of anti-Sp1 antibody (Abcam) was used for supershift assays. The reaction mixture was run on a 6 % polyacrylamide gel/0.5 × Tris-Borate-EDTA (TBE) for 2–3 h. After nylon membrane (GE Healthcare) transfer and UV cross linking, the membranes were incubated with streptavidin-conjugated horseradish peroxidase (HRP), and staining was detected by using the chemiluminescent method.

### 2.13 Chromatin immunoprecipitation (ChIP) assay

ChIP assays were performed according to the manufacturer's instructions (Beyotime). The precipitated DNA was purified by using phenol‒chloroform and subjected to PCR/2 % agarose gel or real-time PCR analysis with primers (Forward:5′- tggggaatggaagaagaggt-3'; Reverse: 5′-ctggccgctgtgaactgcta-3′) for the Sp1-binding site on the MMP2 promoter. DNA isolated before immunoprecipitation was used as an input control, and the data are presented as changes against the input.

### 2.14 Statistical analysis

Data are presented as the mean ± standard error of the mean (SEM) and were statistically analysed by using GraphPad Prism 9 (GraphPad Software, Inc., San Diego, CA). Normal distribution of data was checked by Shapiro‒Wilk test. For normally distributed data, two-group comparisons were performed by using parametric unpaired two-tailed Student's t tests. Comparisons of more than two groups were evaluated via one-way ANOVA with post hoc Tukey's pairwise tests for only one independent variable or via two-way ANOVA with Tukey's correction for two independent variables. The comparisons of AAA incidence and survival rate were conducted with chi-square test and log-rank test, respectively. A *P* value less than 0.05 was considered as statistically significant.

## 3 Results

### 3.1 SMC-specific deletion of Nox1 inhibits AngII-induced abdominal aortic aneurysm

**Fig. 1:**
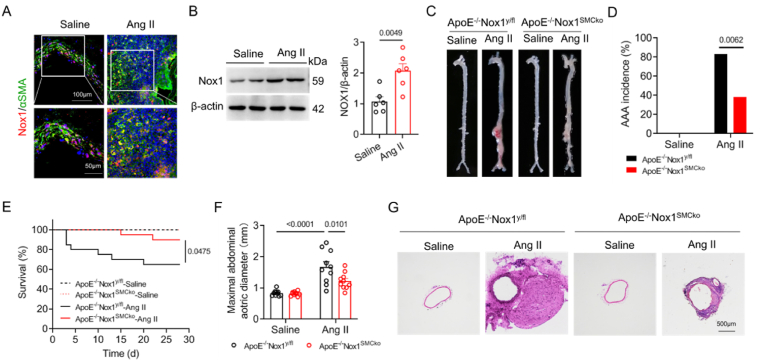
SMC-specific deletion of Nox1 suppresses AAA formation. (A,B) Nox1 expression in AAA tissue from Ang II-induced ApoE^−/−^ mice was detected by using immunofluorescence (yellow) and Western blotting. The data were analysed via Student's *t*-test, n = 6 mice. (C) Representative photographs of whole aorta from ApoE^−/−^Nox1^y/fl^ and ApoE^−/−^Nox1^SMCko^ mice with or without Ang II infusion for 28 days. (D) The incidence of AAA in Ang II-infused ApoE^−/−^Nox1^y/fl^ and ApoE^−/−^Nox1^SMCko^ mice. The data were analysed with the chi-square test; n = 18 mice per group. (E) Survival curves of the indicated groups. The rates were compared by using the log-rank test (n = 20 mice per group). (F) The maximal suprarenal aortic outer diameter in ApoE^−/−^Nox1^y/fl^ and ApoE^−/−^Nox1^SMCko^ mice after Ang II infusion was analysed by using two-way ANOVA with Tukey's test, n = 10 mice per group. (G) Representative images of HE-stained AAA tissue from the indicated mice.

To investigate the expression of Nox1 in aneurysm tissues, we isolated abdominal aortas from ApoE^−/−^ mice that were subcutaneously infused with Ang II for 28 days. The results of fluorescence immunostaining showed that Nox1 expression in SMCs was obviously detected and significantly increased compared with uninfused normal aortas (Fig. 1A). Western blot results further confirmed the increased expression of Nox1 in aortas with aneurysm tissues (Fig. 1B). We generated mice in which Nox1 was SMC-specifically deleted on an ApoE^−/−^ background (ApoE^−/−^Nox1^SMCko^). The expressions of Nox1 in the aorta were verified and showed significantly reduced (Fig. S1). After infusion with Ang II or saline for 28 days, no abnormal aortic morphology or spontaneous abdominal aortic aneurysm (AAA) was found in the saline-treated ApoE^−/−^ Nox1^y/fl^ and ApoE^−/−^Nox1^SMCko^ mice (Fig. 1C). Notably, 38.9 % (7 of 18) of the abdominal aortas in the Ang II-infused ApoE^−/−^Nox1^SMCko^ mice developed aneurysms. The incidence of AAA was significantly lower than that in ApoE^−/−^ Nox1^y/fl^ mice (83.3 %, 15 of 18) (Fig. 1D). Moreover, mortality and maximal aortic diameters were greatly attenuated in mice with SMC-specific Nox1 deletion (Fig. 1E and F). HE staining of the aneurysm sections showed a remarkably reduced aortic well thickness in ApoE^−/−^Nox1^SMCko^ mice compared with ApoE^−/−^ Nox1^y/fl^ mice (Fig. 1G). Together, These data demonstrate that Nox1 deficiency in SMC suppresses AAA formation in mice.

### 3.2 SMC-specific deletion of Nox1 ameliorates the Ang II-induced vascular oxidative stress and inflammation in the aorta

**Fig. 2:**
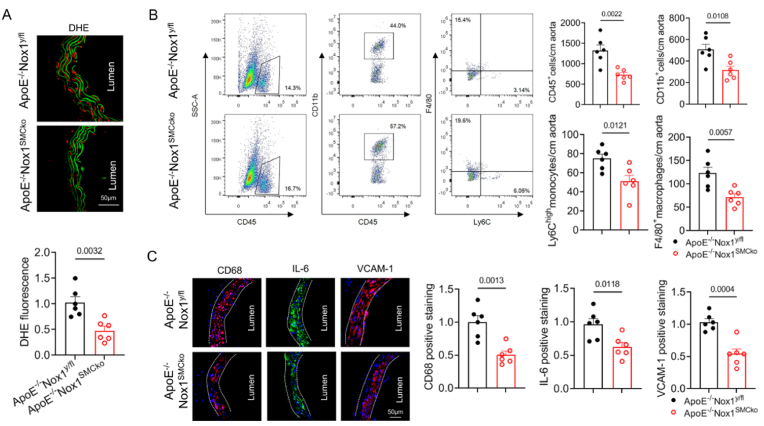
SMC-specific deletion of Nox1 attenuated abdominal aortic ROS and inflammation. The ApoE^−/−^Nox1^y/fl^ and ApoE^−/−^Nox1^SMCko^ mice were infused with Ang II for 28 days. The suprarenal aorta was dissected. (A) The suprarenal aorta was stained with DHE. ROS, as indicated by red staining, were quantified. (B) Immune cell in the suprarenal aorta of Ang II-infused mice was assessed by using flow cytometry. A representative gating strategy is shown, and the numbers of infiltrated cells were quantified. (C) Frozen sections from the abdominal aorta were immunofluorescently stained with anti-CD68, *anti*-IL-6 and *anti*-VCAM-1 antibodies. All of the data were compared by using two-tailed Student's *t* tests (n = 6 mice per group).

As expected, we found that abdominal aortic ROS level was significantly lower in ApoE^−/−^Nox1^SMCko^ mice than in ApoE^−/−^ Nox1^y/fl^ mice after Ang II infusion (Fig. 2A and S2). Flow cytometry analysis revealed a less number of inflammatory leucocytes in abdominal aortas from ApoE^−/−^Nox1^SMCko^ mice, such as CD45^+^ leukocytes, CD45^+^CD11b^+^ myelomonocytic cells, CD11b ^+^ Ly6C^high^ monocytes and CD11b^+^F4/80^+^ macrophages (Fig. 2B). Immunofluorescence staining showed that Nox1 deficiency in SMCs reduced aortic CD68, IL-6 and VCAM-1 expression, thus restraining macrophage adhesion and accumulation, which corroborated the findings from flow cytometry (Fig. 2C). The reduced levels of proinflammatory cytokine IL-6, IL-1β and TNF-α in abdominal aorta of the Nox1 knockout mice were confirmed by Western blotting (Fig. S3). Therefore, SMC-specific deletion of Nox1 attenuates abdominal aortic oxidative stress and inflammation.

### 3.3 Nox1 deficiency in SMCs inhibits ECM degradation in the aorta

**Fig. 3:**
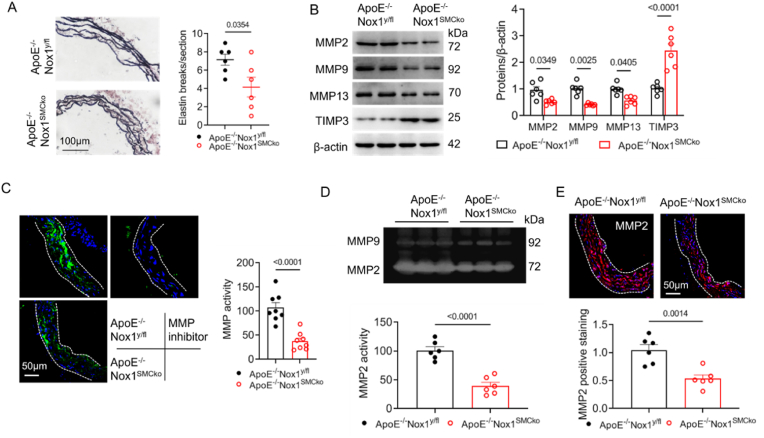
SMC-specific deletion of Nox1 reduced abdominal aorta ECM degradation. The suprarenal aortas were isolated from ApoE^−/−^Nox1^y/fl^ and ApoE^−/−^Nox1^SMCko^ mice after 28 days of Ang II infusion. (A) The aortas were cryo-sectioned and stained with Verhoeff-Van Gieson (VVG). The amount of elastin breaks in the sections was counted. (B) Western blot analysis of protein expression in the abdominal aorta with the indicated antibodies. (C) In situ zymography analysis of the abdominal aorta. The cryosections were incubated with fluorescein-labelled gelatine, and the fluorescence intensity indicates the activity of the MMP. The MMP inhibitor ilomastat was used as a negative control, n = 8 mice/group. (D) Gelatine zymography analysis of the abdominal aorta. (E) The abdominal aortic sections were immunofluorescently-stained with *anti*-MMP2 antibodies. Comparisons were conducted with two-tailed Student's *t* tests (n = 6 mice per group, except in C).

We further examined changes in extracellular matrix components that determine the formation and progression of AAA. Compared with those in ApoE^−/−^ Nox1^y/fl^ mice, reduced elastic fibre fragments and increased elastin expression were detected at abdominal aortas of ApoE^−/−^Nox1^SMCko^ mice (Fig. 3A,S4A). Nox1 SMC-specific depletion restored the balance between MMPs and tissue inhibitors of metalloproteinases (TIMPs), in which the expressions of MMP2, MMP9 and MMP13 decreased and that of TIMP1 and TIMP3 but not TIMP2 increased (Fig. 3B–S4B). In situ zymography analysis by fluorescein-conjugated gelatine showed that the proteolytic activity of the aortic MMP was 73 % lower in ApoE^−/−^Nox1^SMCko^ mice than in ApoE^−/−^Nox1^y/fl^ mice (Fig. 3C). A 61 % reduction in MMP2 gelatinolytic activity was also detected in aortas isolated from Nox1 SMC-specific deficient mice via a gelatine zymography assay (Fig. 3D). Consistent with these findings, immunofluorescence results showed the MMP2 level in the abdominal aorta was reduced by 47 % in Nox1 knockout mice (Fig. 3E). Overall, SMC-specific deletion of Nox1 reduces ECM degradation in the abdominal aorta.

### 3.4 Nox1 interacts with PAK1 in Ang II-induced VSMCs

**Fig. 4:**
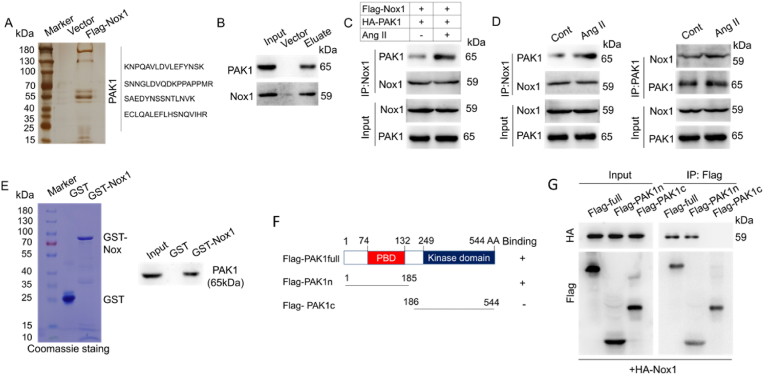
Nox1 interacts with PAK1 in Ang II-induced VSMCs. (A) VSMCs were transfected with Flag-Nox1 and stimulated with Ang II (1 μM). After affinity purification, the Nox1-binding proteins were separated, silver-stained, and subjected to mass spectrometry analysis. (B) The eluates were immunoblotted with *anti*-PAK1 and *anti*-Nox1 antibodies. (C) Ang II-treated VSMCs were cotransfected with Flag-Nox1 and HA-PAK1. The association between Flag-Nox1 and HA-PAK1 was detected via coimmunoprecipitation. (D) Coimmunoprecipitation was used to detect the endogenous binding of Nox1 and PAK1 in Ang II-induced VSMCs. (E) Purified Flag-PAK1 was incubated with GST or recombinant GST-Nox1, and the complexes were subjected to a pull-down assay. (F,G) Schematic diagram of the PAK1 protein domains. Cells were cotransfected with HA-Nox1 and truncated Flag-PAK1s, and the domain of interaction with Nox1 was detected by using immunoprecipitation. The experiments were performed at least three times.

To explore the molecular basis underlying the effects of Nox1 on vascular remodelling, we carried out affinity purification and analysed Nox1-interacting proteins. VSMCs were stably transfected with Flag-Nox1 and stimulated with Ang II. After being immunopuerified with an anti-Flag column, the bound proteins were resolved via PAGE and silver stained (Fig. 4A). Mass spectrometry indicated that PAK1 is potentially associated with Nox1 (Fig. 4A). The presence of PAK1 in the Nox1-containing complex was verified by immunoblotting the column eluates (Fig. 4B). Coimmunoprecipitation assays also confirmed the association of Nox1 and PAK1 in vivo (Fig. 4C and D). Furthermore, glutathione S-transferase (GST)-fused Nox1 was incubated with Flag-PAK1. PAK1 was successfully pulled down, suggesting the in vitro binding of PAK1 with Nox1 (Fig. 4E); moreover, the amino-terminal fragment containing the PBD domain responded to the interaction (Fig. 4F and G).

### 3.5 Nox1 and ROS increase PAK1 activation in Ang II-induced VSMCs

**Fig. 5:**
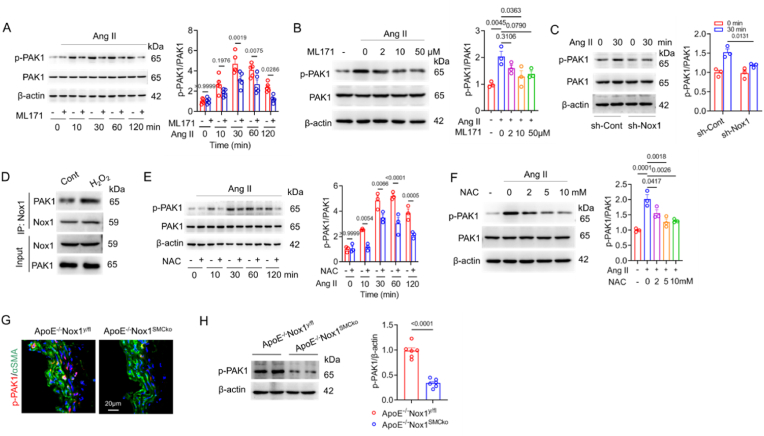
Nox1 and ROS increase PAK1 activation. (A) The *p*-PAK1 level in Ang II (1 μM)-induced VSMCs treated with or without the Nox1 inhibitor ML171 (10 μM) for the indicated period of time was determined by using Western blotting. Data were analysed by using two-way ANOVA, n = 5. (B) *p*-PAK1 levels in cells incubated with Ang II and indicated concentrations of ML171 for 30 min. Data were analysed by using one-way ANOVA, n = 3. (C) VSMCs were incubated with Nox1-specific shRNA (sh-Nox1), and *p*-PAk1 was detected by using immunoblotting 30 min after Ang II induction. Two-way ANOVA, n = 3. (D) Co-IP assay for the association of Nox1 and PAK1 in cells stimulated with H_2_O_2_. VSMCs were incubated with H_2_O_2_ (150 μmol/L) for 24 h. (E) *p*-PAK1 levels in VSMCs exposed to NAC (5 mM) and Ang II for the indicated times. Two-way ANOVA, n = 3. (F) Ang II-induced *p*-PAK1 in the presence of NAC at indicated concentrations for 30 min. One-way ANOVA, n = 3. (G,H) Immunofluorescence and immunoblot staining with an *anti*-*p*PAK1 antibody in the suprarenal aortas of ApoE^−/−^Nox1^y/fl^ and ApoE^−/−^Nox1^SMCko^ mice 28 days after Ang II infusion. Student's *t*-test, n = 6.

Subsequently, we investigated whether PAK1 activation was controlled by Nox1. VSMCs were stimulated with Ang II and with or without the Nox1 inhibitor ML171 for different periods of time. We found that the level of phosphorylated PAK1 (*p*-PAK1) significantly increased within 10–120 min by Ang II and was significantly inhibited by ML171 treatment (Fig. 5A). ML171 dose-dependently inhibited Ang II-induced PAK1 activation (Fig. 5B). The *p*-PAK level was also reduced in cells with genetic knockdown of Nox1 (Fig. 5C). Nox1 is a main isoform of ROS-generating enzymes in SMCs. We then investigated whether the activation of PAK1 by Ang II is dependent on increasing intracellular ROS. We found that H_2_O_2_ treatment strikingly enhanced the association of Nox1/PAK1 (Fig. 5D). Treatment with the ROS scavenger N-acetylcysteine (NAC) decreased *p*-PAK1 level (Fig. 5E and F). Finally, double immunofluorescence staining showed that VSMC-*p*-PAK1 was greatly lower in ApoE^−/−^Nox1^SMCko^ mice than in ApoE^−/−^ Nox1^y/fl^ mice, and a >60 % reduction in aortic *p*-PAK1 was detected by Western blotting (Fig. 5G and H). These data demonstrate that Ang II stimulates Nox1/ROS and increases PAK1 activation.

### 3.6 Nox1 promotes SMC proliferation, migration and differentiation by activating PAK1

**Fig. 6:**
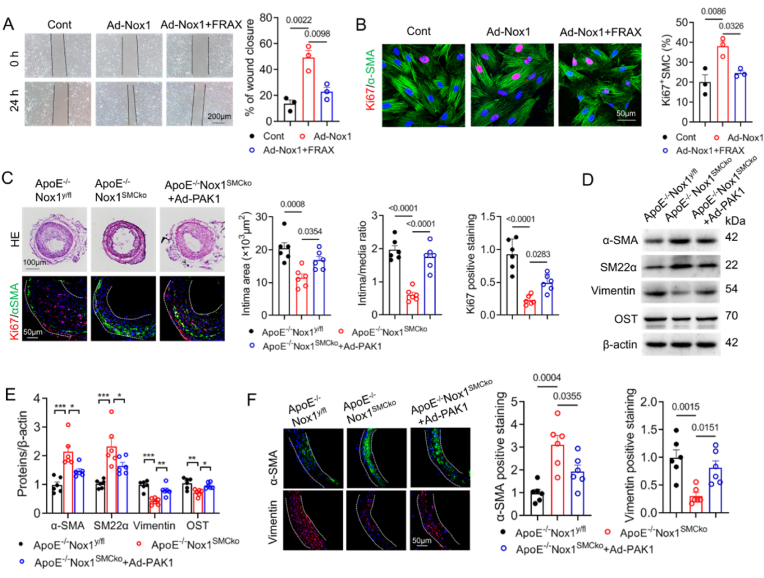
Nox1/PAK1 regulates VSMC proliferation, migration and differentiation. (A) Cell scratch assay for VSMC migration. VSMCs were transfected with Ad-Nox1 and incubated with or without 1 μM FRAX597 for 24 h n = 3. (B) Representative immunofluorescence staining with an *anti*-Ki67 antibody (red) and an *anti*-α-SMA antibody (green). Nuclei were counterstained with DAPI (blue). n = 3. (C) Neointimal formation in the carotid artery induced by ligation injury for 14 days in ApoE^−/−^Nox1^y/fl^, ApoE^−/−^Nox1^SMCko^ and ApoE^−/−^Nox1^SMCko^ + Ad-PAK1 mice. Representative HE and immunofluorescence images of Ki67 (red), α-SMA (green) and DAPI (blue) staining. The intimal area, intima/media ratio and relative Ki67-positive staining were quantified. n = 6. (D–F) Immunoblot and immunofluorescence analysis of the expression of VSMC contractile and synthetic proteins in the suprarenal aorta. n = 6. ∗*P* < 0.05, ∗∗*P* < 0.01, ∗∗∗*P* < 0.001. All of the data were analysed via one-way ANOVA with a post hoc Tukey's test.

High level of PAK1 expression was detected in the Ang II-induced aneurysmal tissues of ApoE^−/−^ mice (Figs. S5A and B). We subsequently speculated that Nox1-regulated PAK1 is involved in vascular disorders. To test the hypothesis, we firstly determined the role of Nox1/PAK1 in VSMC activation. VSMCs were transfected with Ad-Nox1, and incubated with the PAK1 inhibitor FRAX597 for 24 h. The specifity of PAK1 inhibition by the FRAX597 was verified by Western blot (Fig. S6). Wound healing assay showed the Nox1 overexpression promoted VSMC migration, which was suppressed by FRAX597 (Fig. 6A). The results of Ki67 immunostaining suggested that VSMC proliferation was induced by Nox1 and inhibited after FRAX597 treatment (Fig. 6B). To investigate the involvement of PAK1 in Nox1-induced VSMC activation in vivo, we assessed neointima hyperplasia after carotid artery ligation injury. We found that the pronounced neointima formation induced by injury was significantly suppressed in the mice of ApoE^−/−^Nox1^SMCko^ in comparison with the ApoE^−/−^ Nox1^y/fl^ mice, as evidenced by the decreased intima area and intima/media ratio (Fig. 6C). However, PAK1 overexpression substantially increased neointimal hyperplasia in ApoE^−/−^ Nox1^SMCko^ mice at 14 d after carotid artery ligation (Fig. 6C). As expected, Ki67 expression in the injury-induced artery was inhibited by Nox1 SMC-specific deletion and was also restored by PAK1 overexpression (Fig. 6C). Therefore, these data suggested that the increase in injury-induced SMC proliferation induced by Nox1 required PAK1. Moreover, the expressions of SM22α and α-SMA were increased, whereas the levels of osteopontin and vimentin were reduced in the carotid artery after ligation in ApoE^−/−^Nox1^SMCko^ mice, thus suggesting that Nox1 deletion inhibited VSMC phenotypic switching (Fig. 6D–F). While, the change in VSMC phenotype was reversed by PAK1 overexpression (Fig. 6D–F). The Nox1/PAK1-mediated VSMC phenotype transition was also observed to occur in Ang II-induced aortas (Fig. S7). Overall, Nox1/PAK1 is involved in regulation of VSMC migration, proliferation and differentiation.

PAK1 has been reported to regulate vimentin and is involved in cell cytoskeleton remodelling [13], and the change of cytoskeleton is closely related to VSMC migration. We then investigated whether Ang II/Nox1 modulated cell cytoskeleton via PAK1 regulation. The VSMCs were incubated with Ang II, and with or without the Nox1 inhibitor ML171 or the PAK1 inhibitor FRAX597. Results showed that Ang II significantly induced vimentin phosphorylation (*p*-vimentin) and inhibited phosphorylated cofilin (*p*-cofilin) (Fig. S8A). However, changes in the phosphorylation levels were not observed after the addition of ML171 or FRAX597 (Fig. S8A). Rhodamine-phalloidin staining revealed that ML171 or FRAX597 treatment reduced Ang II-induced filamentous actin and cell extension, as indicated by less intense staining and a more rounded morphology compared with Ang II -treated cells (Fig. S8B). Moreover, we found the PAK1 overexpression reversed the effects of ML171 on *p*-vimentin, *p*-cofilin and filamentous actin (Fig. S8C). These data suggest that Nox1/PAK1 mediate the Ang II-induced cytoskeleton modulation, which may facilitates the VSMC migration.

Finally, to further explore the signalling pathway involved in Nox1/PAK1-mediated VSMC activation, we incubated Ang II-treated VSMCs with ML171 or FRAX597. Immunoblotting demonstrated that three MAPKs (ERK1/2, p38 and JNKs) were activated by Ang II and were significantly reduced by ML171 or FRAX597, while increased by PAK1 overexpression (Figs. S9A–C). These data suggested that MAPKs is downstream of Nox1/PAK1, and the signalling cascade affects VSMC activation.

### 3.7 Nox1/PAK1 promotes NF-κB activation and the inflammatory response in Ang II-stimulated SMCs

The above mentioned findings have demonstrated that SMC-specific deletion of Nox1 causes amelioration in Ang II-induced vascular inflammation. The NF-κB activity also controlled by MAPKs upon Ang II stimulation (Fig. S10A). Therefore, we set out to explore the effects of Nox1/PAK1 on NF-κB activation in VSMCs. We found that Ang II expectedly increased *p*-p65 and *p*-IkBα, whereas this increase was inhibited by ML171 or FRAX597 (Fig. S10B). ML171 and FRAX597 also reduced the nuclear accumulation of p65, thus suggesting the inhibition of p65 nuclear translocation (Figs. S10C and D). This finding was further confirmed by immunofluorescence staining, in which nuclear p65 was dramatically reduced in cells exposed to ML171 or FRAX597 (Fig. S10E). The EMSA results showed that the two inhibitors reduced p65 DNA binding activity in the nucleus (Fig. S10F). Specific binding was detected via supershift EMSA by using p65 antibodies (Fig. S10F). Consequently, the AngII-induced IL-6 transcription was found significantly reduced in ML171 or FRAX597 treated VSMCs (Fig. S10G). Finally, co-treatment of ML171 and adenovirus-mediated PAK1 expression vector (Ad-PAK1) drametically increased *p*-p65, nuclear p65 and IL-6 expression compared with treatment with ML171 alone (Figs. S10H–J). Therefore, these data demonstrated that Ang II-activated NF-κB was suppressed by Nox1/PAK1 inhibition and suggested that the NF-κB was involved in the Nox1/PAK1-mediated inflammatory response.

### 3.8 Nox1/PAK1 upregulates Sp1-mediated MMP2 expression in Ang II-induced VSMCs

**Fig. 7:**
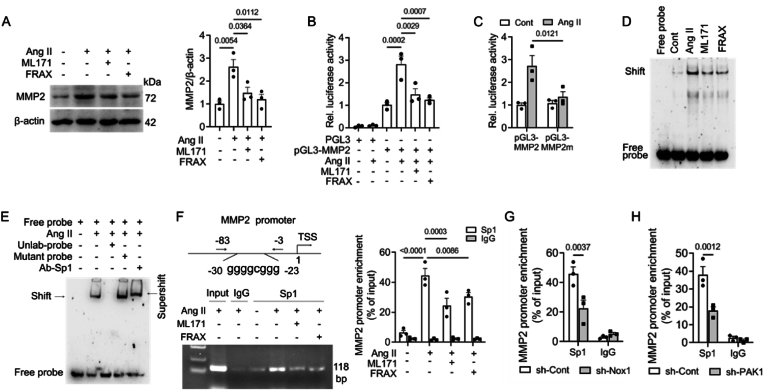
Nox1/PAK1 increases MMP2 expression by activating Sp1 in AngII-induced VSMCs. (A) Immunoblotting detection of MMP2 expression in cells treated as indicated for 24 h. (B) Luciferase assay of MMP2 promoter activity in cells subjected to the indicated treatments for 24 h. (C) Luciferase activity of MMP2 promoter mutants in Sp1 binding sites ranging from −30 to −23 bp in length in cells treated with Ang II for 24 h. (D) EMSA for the in vitro binding of Sp1 to the MMP2 promoter. Cells were treated with Ang II for 24 h, and nuclear extracts were incubated with a probe containing the Sp1 binding site in the MMP2 promoter. (E) Competitive and supershift EMSAs indicated the presence of the probe and Sp1 in the binding complex. (F) The Sp1 binding site in the MMP2 promoter and the binding of Sp1 were analysed by using ChIP. (G,H) ChIP assay for the binding of Sp1 to the MMP2 promoter in cells with Nox1 or PAK1 knockdown. One-way ANOVA with Tukey's test in A and B and two-way ANOVA with Tukey's test in C, F, G and H, n = 3.

The above in vivo data have demonstrated that the levels of the matrix metalloproteinases MMP2, MMP9 and MMP13 were reduced in Nox1 SMC-specific knockout aortas. We then further tested whether the expression of these MMPs is controlled by Nox1/PAK1. VSMCs were stimulated with Ang II and treated with ML171 or FRAX597. Immunoblotting showed that the MMP2 levels were strikingly increased by Ang II and decreased by either inhibitor (Fig. 7A). We further investigated the transcriptional regulation of MMP2. VSMCs were transfected with a vector expressing luciferase driven by the MMP2 promoter. The results showed that Ang II-induced luciferase activity was inhibited by ML171 or FRAX597, thus suggesting a reduction in the transcriptional activity of the MMP2 promoter (Fig. 7B). The MMP2 promoter contains binding sites for multiple transcription factors, including E2F1, AP-1, STAT1 and Sp1. We also found that the Sp1 activity was modulated by Nox1/PAK1 and MAPKs (Figs. S11A and B). Therefore, we subsequently further assessed the functional role of Sp1 in Nox1/PAK1-mediated MMP2 transcriptional control. A luciferase reporter vector harbouring the MMP2 promoter with deletion of the Sp1 binding site from −30 to −23 bp was transfected into VSMCs, and the luciferase activity was significantly reduced (Fig. 7C). Results of EMSA showed ML171 and FRAX597 treatments reduced the Ang II-induced direct binding of the Sp1 to MMP2 promoter (Fig. 7D and E). Moreover, ChIP result further confirmed the reduced binding of Sp1/MMP2 promoter in cells treated with ML171 or FRAX597 (Fig. 7F). Similar results were obtained in cells transfected with sh-RNAs specific for Nox1 or PAK1 (Fig. 7G and H). Therefore, Sp1 is directly involved in Nox1/PAK1-reglated MMP2 transcription.

### 3.9 Overexpression of PAK1 aggravates AAA and vascular inflammation in Nox1-deficient mice

**Fig. 8:**
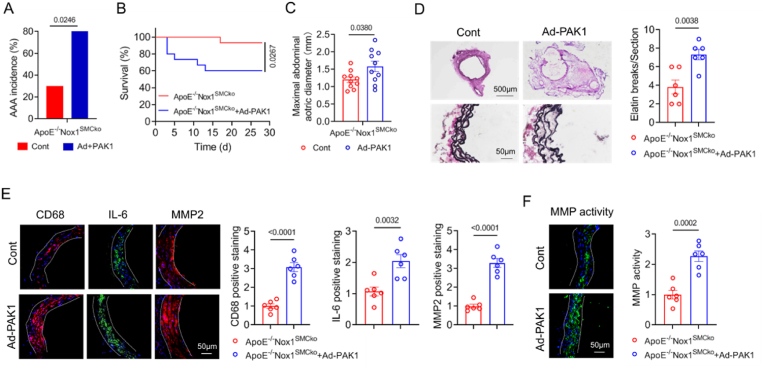
PAK1 overexpression exacerbates AAA formation. ApoE^−/−^Nox1^SMCko^ mice were administered Ad-PAK1 and infused with Ang II for 28 days. (A–C) PAK1 overexpression increased AAA incidence (chi-square test, n = 10), survival rate (log-rank test, n = 15) and maximal suprarenal aorta width (Student's *t*-test, n = 10). (D) Representative images of cross-sections stained with HE and VVG, and the numbers of elastin breaks were counted. (E) Representative images of immunofluorescence staining for CD68, IL-6 and MMP2 in the abdominal aorta. Positive staining was quantified. (F) Cross-sections of the abdominal aorta were incubated with fluorescein-gelatine, and the MMP activity was assessed by using in situ zymography. Student's *t*-test in D-F, n = 6.

Given that PAK1 has been established to be involved in Ang II/Nox1-induced VSMC activation, inflammation and MMP2 expression, we further investigated the effects of PAK1 overexpression on AAA formation and vascular inflammation in mice with Nox1 SMC-specific deletion. The ApoE^−/−^Nox1^SMCko^ mice were infected with Ad-PAK1, and were infused with Ang II for 28 d. We found that the overexpression of PAK1 increased the incidence of AAA by 1.6-fold (30 % vs. 80 %) and maximal abdominal aortic diameter, and significantly reduced survival (Fig. 8A–C). Aneurysm sections were stained with HE and VVG, and the results showed that PAK1 overexpression increased the thickness of the aortic wall and promoted elastic fibre degradation (Fig. 8D). Moreover, we found that PAK1 overexpression increased abdominal aortic CD68, IL-6, MMP2 and gelatinolytic activity (Fig. 8E and F). These data demonstrated that increasing PAK1 promotes vascular inflammation, ECM remodelling and AAA progression, and suggested that the effects of Nox1 on AAA are dependent on PAK1 activation.

## 4 Discussion

**Fig. 9:**
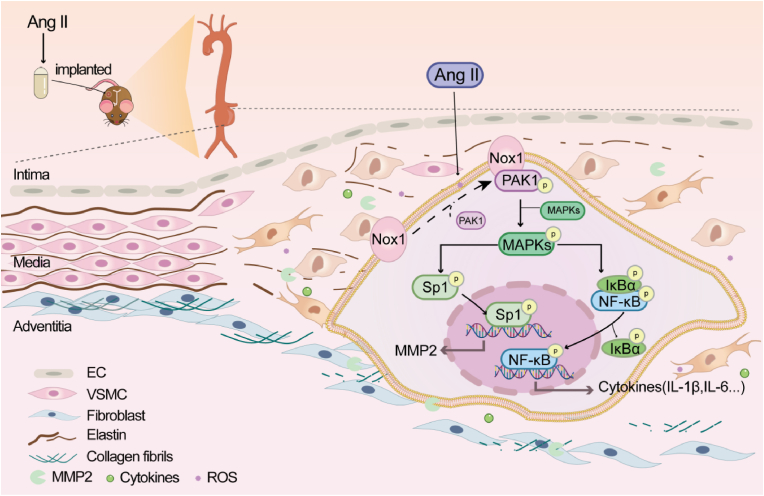
Schematic illustration of the effects of Nox1 in AAA formation.

Current studies provide the evidence that SMC-specific deletion of Nox1 alleviates Ang II-induced vascular inflammation, ECM degradation and restrains AAA formation in ApoE^−/−^ mice. We find that Nox1 interacts with and activates PAK1 in SMCs, which causes SMC migration, proliferation, differentiation and cytoskeletal changes in response to Ang II stimulation. The Ang II-induced Nox1/PAK1 increases MAPKs and NF-κB activation and Sp1-mediated MMP2 expression. The protective effects of Nox1-deficient on AAA development were reversed by PAK1 overexpression. Therefore, these compelling findings establish a prominent role of Nox1 in vascular remodelling and AAA formation, as illustrated in Fig. 9.

Among the seven types of NADPH oxidase, the Nox1, Nox2 and Nox4 isoforms are found in the vascular wall. The Nox1 is strongly expressed in VSMCs and relatively less expressed in endothelial cells than Nox2 and Nox4. Although both Nox1 and Nox4 are expressed in VSMCs, Nox4 is constitutively active, whereas Nox1 is induced by the pressor Ang II, cytokines (PDGF, IL-1β and TNF-α) and thrombin in VSMCs [[28], [29], [30], [31], [32]]. Nox1 profoundly affects SMC migration, proliferation, and phenotypic modification and is thereby implicated in vascular remodelling in response to such stimulators [[18], [19], [20],[33], [34], [35]]. It has been reported that Nox1 overexpression in SMCs augments Ang II-induced hypertension and smooth muscle hypertrophy; moreover, the deletion of Nox1 alleviates elevated blood pressure and maintains endothelium-dependent vascular relaxation in Ang II-infused mice [21,22]. Recently, the downregulation of Nox1 was found to inhibit KLF4-mediated phenotypic change of SMC to macrophage-like cells and increase atherosclerotic plaque stability [36]. Therefore, these evidences indicate that Nox1 modulates SMC activation and then promotes the progression of vascular diseases. Herein, we extend this notion and provide data suggesting that Nox1 induced by Ang II increase SMC proliferation, migration and phenotypic modification, which potentiates AAA formation.

Nox1, a membrane-bound enzyme, is activated by various extracellular stimulators. Although Nox1 has been demonstrated to profoundly affect vascular pathologies, its role in intracellular signalling events is not well understood. By using affinity purification and mass spectrometry, we identified PAK1 as a downstream effector that is regulated by Nox1/ROS during AAA formation under Ang II stimulation. Our results are similar to previous findings that Nox1 deficiency decreases PAK1 expression in VSMCs and that Y-27632 increases PAK1 phosphorylation by Nox1, leading to neurite outgrowth in PC12 cells [18,37]. Furthermore, we provide evidence that Nox1 directly binds to PAK1 and that this interaction activates PAK1 and the downstream MAPK pathway. Thus, our findings help to further elucidate Nox1-mediated cellular signalling. In addition, we found that Nox1 inhibition and treatment with the ROS scavenger NAC inhibited PAK1 activation, which provided the direct evidence and demonstrated that PAK1 is a redox-sensitive enzyme controlled by the intracellular ROS level. This scenario is supported by the fact that PAK1 phosphorylation can be modified by 3-phosphoinositide-dependent kinase-1 (PDK1), whereas PDK1 has been reported to be induced by oxidative stress [38,39,41].

However, PAK1 can be activated by PDK1 kinase [39], PI3K/AKT [40,42] and lipids such as sphingosine [43] and phosphoinositides [44] in a GTPase-independent manner, especially when PAK1 is recruited to the cell membrane. It is well accepted that the activation of PAK1 requires the binding of GTP-bound Rac1 and CDC42, thus causing autophosphorylation and conformational changes, which subsequently increases catalytic activity. Rac1 is a component of the Nox1 complex, which binds and activates Nox1, as well as its regulatory subunit NoxA 1. Moreover, Nox1 is a membrane-bound molecule that recruits and associates with PAK1 in the cell membrane after Ang II- stimulation in our study. Therefore, whether Nox1-mediated PAK1 activation requires Rac1 or other signalling molecules remains to be further investigated.

The vascular ECM that composed of elastin, collagens, fibronectin and laminin, provides mechanical support to the vessel wall. Progressive degradation of ECM components contributes to AAA formation. MMPs are the main factors involved in ECM protein hydrolysis. The activities of the MMP are regulated by a series of TIMPs that exhibit high affinity and direct binding with MMPs. The MMP/TIMP balance is crucial in maintaining ECM homeostasis. Disturbance of the balance caused by proinflammatory cytokines or oxidative stress has been observed in various diseases and cell processes, including cancer progression and metastasis, as well as atherosclerotic plaque rupture. MMP2/9 are upregulated in AAA in both mice and patients [[45], [46], [47], [48]]. Herein, we demonstrated that SMC-specific deletion of Nox1 reduces the aortic proteolytic activity of MMPs, as assessed by using in situ zymography. In particular, we provide further evidence that Ang II-stimulated Nox1 promotes Sp1-mediated MMP2 transcription. However, clinical and animal model studies have demonstrated that MMP8 and MMP9 is closely related to AAA formation [49,50]; moreover, TIMP1-3 has been shown to protect against the development of experimental AAA [[51], [52], [53]]. Therefore, our results do not preclude the possibility that other molecules involved in ECM modifications are also regulated by the Nox1/PAK1 axis.

Leucocyte endothelial infiltration increases expressions of pro-inflammatory cytokine and triggers inflammatory response. On the other hand, SMCs can be activated and transformed into macrophage-like cells, which have been demonstrated to also contribute to vascular inflammation [54,55]. Our flow cytometry results showed Nox1 deletion reduced aortic CD45^+^ cells (Fig. 2B). Further, we obsearved that positive staining of macrophage marker CD68 largely appeared at the media (Fig. 2C). Based on the fact that Nox1 knockout reduced SMC phenotypic transition, as demonstrated in Fig. 6F, the Nox1-mediated vascular inflammation may be caused by both monocyts/macrophages infiltration and phenotypic transformation.

Vascular inflammation is a driving force of AAA formation. The infiltrated and transformed inflammatory cells secrete cytokines and proteinases, thus leading to vascular remodelling. Herein, we found that Nox1 deficiency in SMCs reduced the immune cells, and expressions of the proinflammatory cytokines in aortas and ameliorating vascular inflammation. Atherosclerosis has been considered as a chronic inflammatory disease [54]. Excess lipid deposited in the vessel wall induces macrophage adhesion, migration and infiltration across the endothelium, which correspondingly increases the inflammatory response [56]. In addition, the MMP upregulation and ECM disruption lead to plaque rupture in later stages [57,58]. Therefore, it is reasonable to expect that Nox1/PAK1 may contribute to the initiation and progression of atherosclerosis.

In conclusion, we demonstrated that SMC-specific Nox1 deficiency ameliorates vascular inflammation and restores the structural integrity of the aortic wall, thus inhibiting AAA formation in Ang II-infused mice. Mechanistically, Ang II increases the association of Nox1 with PAK1 to activate SMCs and upregulate proinflammatory cytokines and MMP2. Herein, we highlight the determinant role of Nox1/PAk1 in ECM and vascular remodelling. Our data suggest that the inhibition of Nox1/PAK1 may be a promising strategy for preventing AAA.

## CRediT authorship contribution statement

**Hui He:** Methodology, Investigation, Data curation. **Tianyu Jiang:** Methodology, Investigation, Data curation. **Meng Ding:** Methodology, Investigation, Data curation. **Yuan Zhu:** Methodology, Investigation, Data curation. **Xiaoting Xu:** Methodology, Investigation, Data curation. **Yashuang Huang:** Methodology, Investigation, Data curation. **Wenfeng Yu:** Funding acquisition. **Hailong Ou:** Writing – review & editing, Writing – original draft, Project administration, Methodology, Funding acquisition, Formal analysis, Conceptualization.

## Funding

This work was supported by the National Natural Science Foundation of China (no. 32060219, 32260232, and 82060232); Guizhou Provincial Science and Technology Projects (no. [2022]038); Special Grant for Central Government Supporting Local Science and Technology Development, Science and Technology Department of Guizhou Province, China (no. [2019] 4008), and Science and Technology Plan Project of Guizhou Province, China (no. [2020]1Z060).

## Declaration of competing interest

none.

## Data Availability

Data will be made available on request.

## Abbreviations

Nox1: NADPH oxidase 1
AAA: Abdominal aortic aneurysm
HFD: High-fat-diet
VSMC: Vascular smooth muscle cell
PAK1: p21-activated kinase 1
ECM: Extracellular matrix
MMP2: Matrix metalloproteinase 2
