# Supplementary material for: Nox1/PAK1 is required for angiotensin II-induced vascular inflammation and abdominal aortic aneurysm formation
Source: Redox Biol. 2024 Dec 19;79:103477. doi: 10.1016/j.redox.2024.103477 (PMC11732235; doi:10.1016/j.redox.2024.103477)
Supplement: Multimedia component 1 [file mmc1.docx]

**Supplementary materials**

**
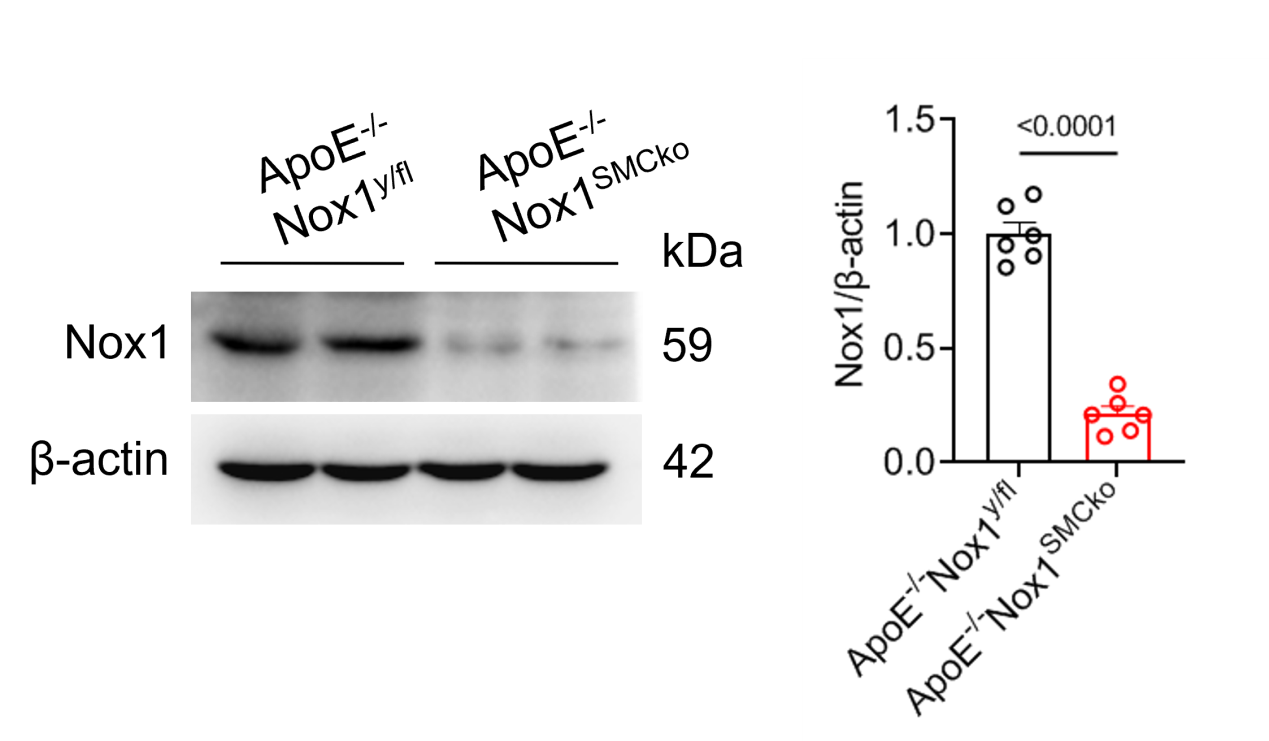
**

**Fig.S1. Nox1 expression in aortas isolated from ApoE^-/-^Nox1^y/fl^ and ApoE^-/-^Nox1^SMCko^ mice.**

Aorta from 6-week-old male mice were homogenized and subjected to Western blot. Student's *t*-test, n=6.

**Fig.S2. Determination of ROS level in abdominal aorta.**

The ApoE^-/-^Nox1^y/fl^ and ApoE^-/-^Nox1^SMCko^ mice were infused with Ang II for 28 days. Abdominal aortas were isolated and enzymatically digested with lysis buffer containing collagenase at 37 °C for 1 h, and passed through a 70 μm cell strainer. The single-cell suspensions were incubated with 10 μmol/L DCFH-DA for 2 h at 37 °C. The intensities of DCF were measured on fluorescence microplate reader with wavelengths of excitation and emission at 488/520. Student's *t*-test, n=6.

**
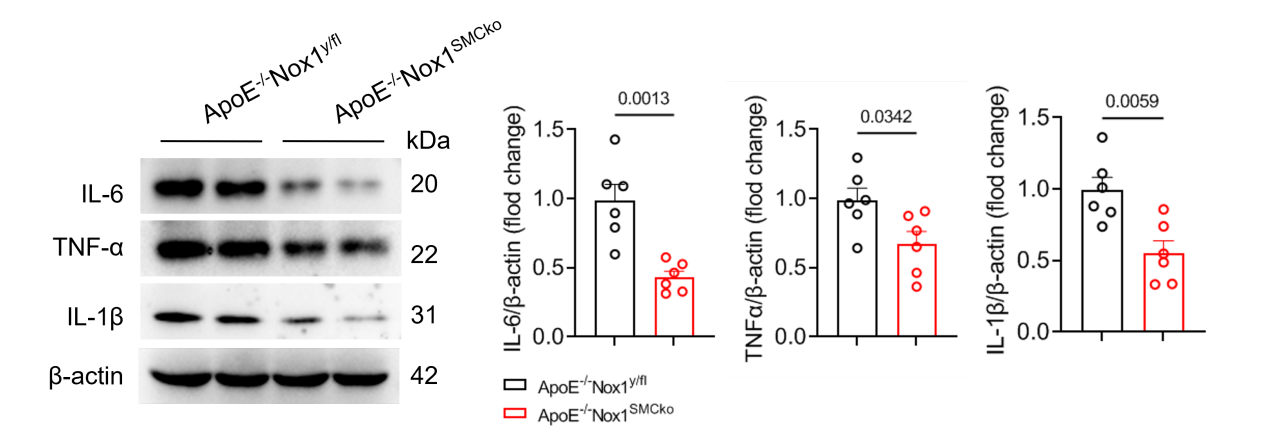
**

**Fig.S3. Expressions of pro-inflammatory cytokine in abdominal aorta.**

The ApoE^-/-^Nox1^y/fl^ and ApoE^-/-^Nox1^SMCko^  mice were infused with Ang II for 28 days. Abdominal aortas were homogenized for Western blot with antibodies against IL-6, IL-1β and TNF-α. Student's *t*-test, n=6.

**
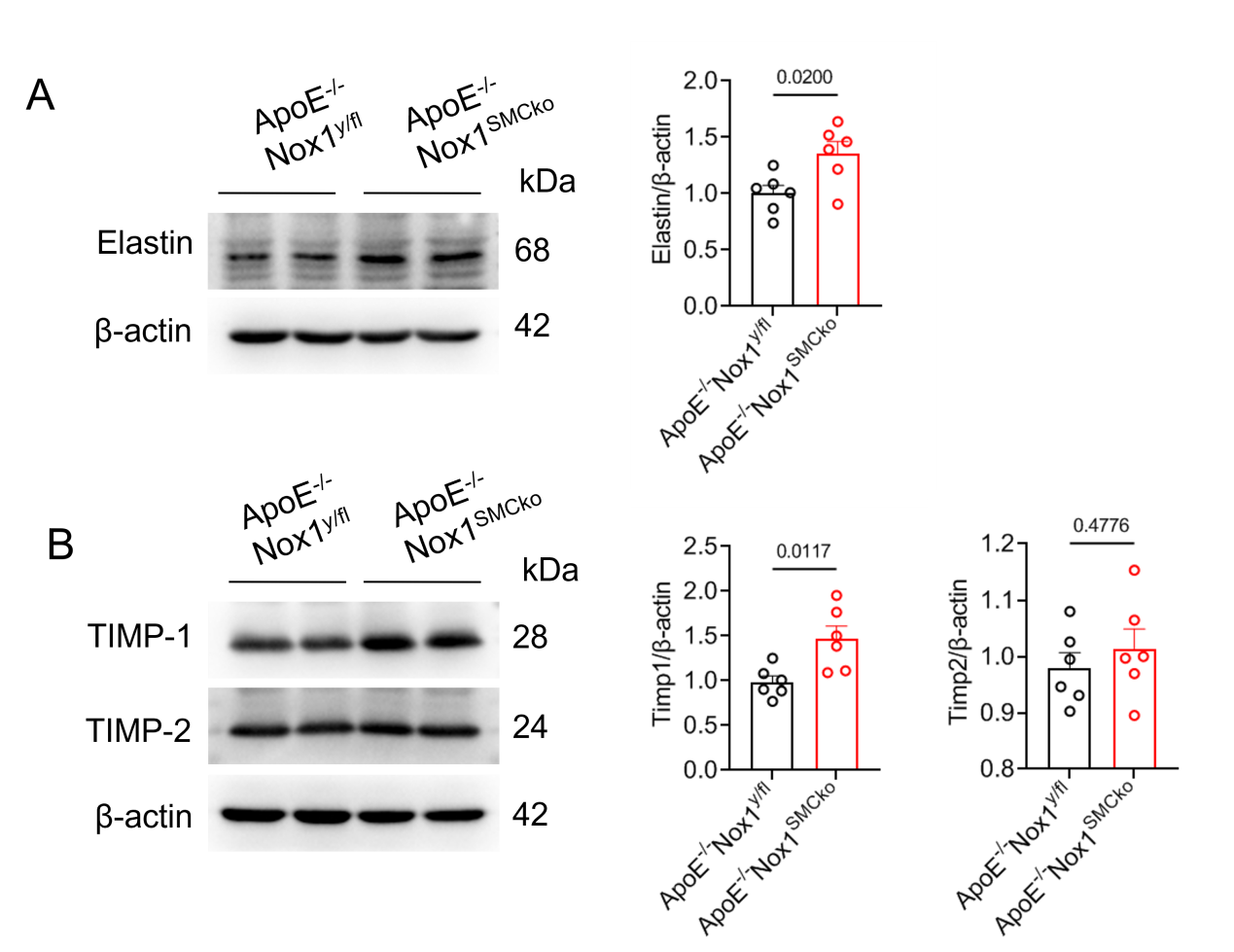
**

**Fig.S4. Expressions of ECM components in abdominal aortas isolated from ApoE^-/-^Nox1^y/fl^ and ApoE^-/-^Nox1^SMCko^ mice.**

Abdominal Aorta from AngII-infused mice were homogenized and subjected to Western blot. Student's *t*-test, n=6.

**
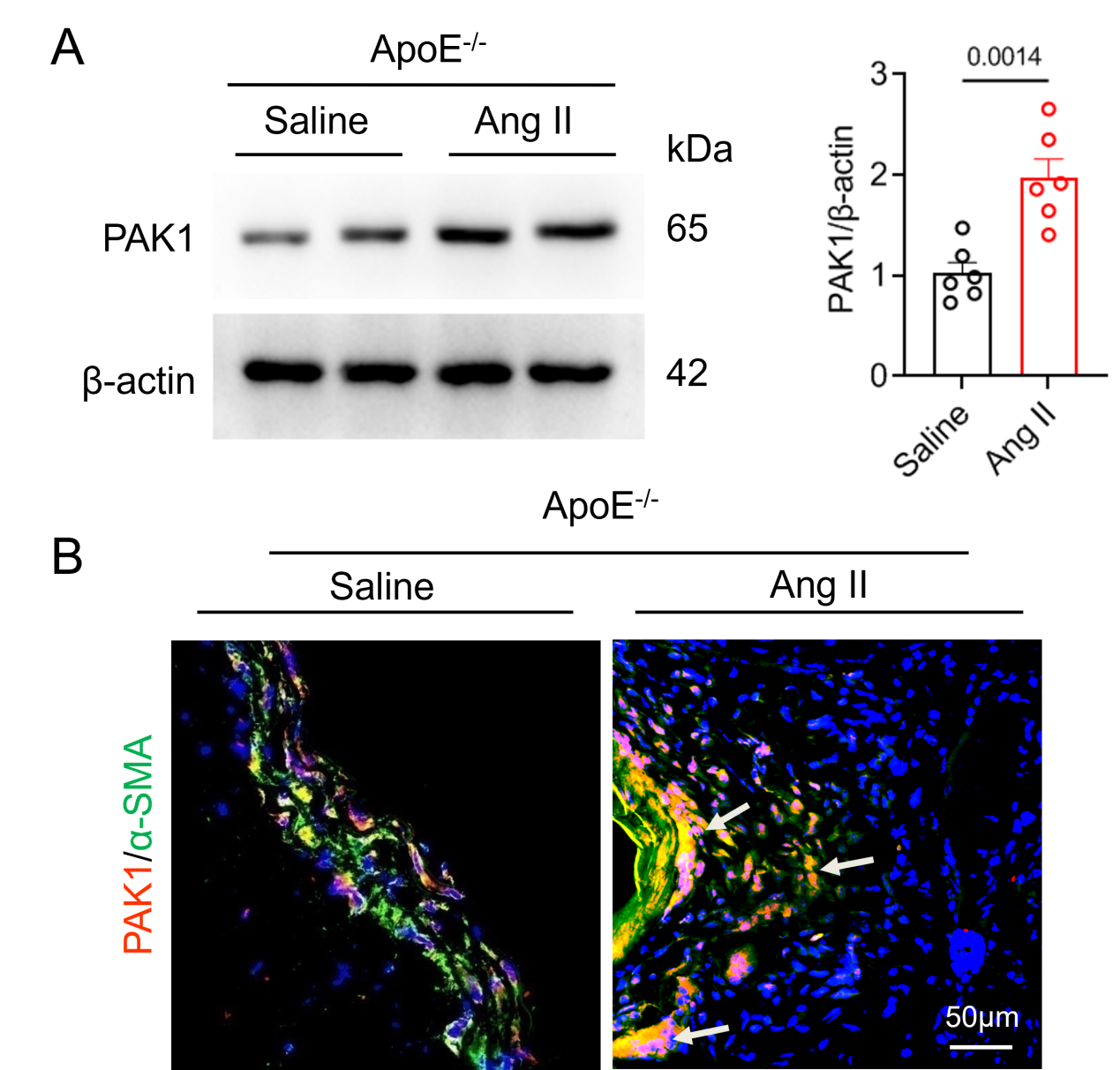
**

**Fig. S5. PAK1 expression in AAA.**

(A) Western blot analysis of PAK1 expression in abdominal aorta from ApoE^-/-^ mice infused with saline or Ang II for 28 days. Student's *t*-test, n=6. (B) The AAA from saline or Ang II-infused ApoE^-/-^ mice were cross-sectioned. Representative immunofluorescent stainings of PAK1 (red), SMA (green) (arrow). Nuclei were counterstained with DAPI (blue). Student's *t*-test, n=6.


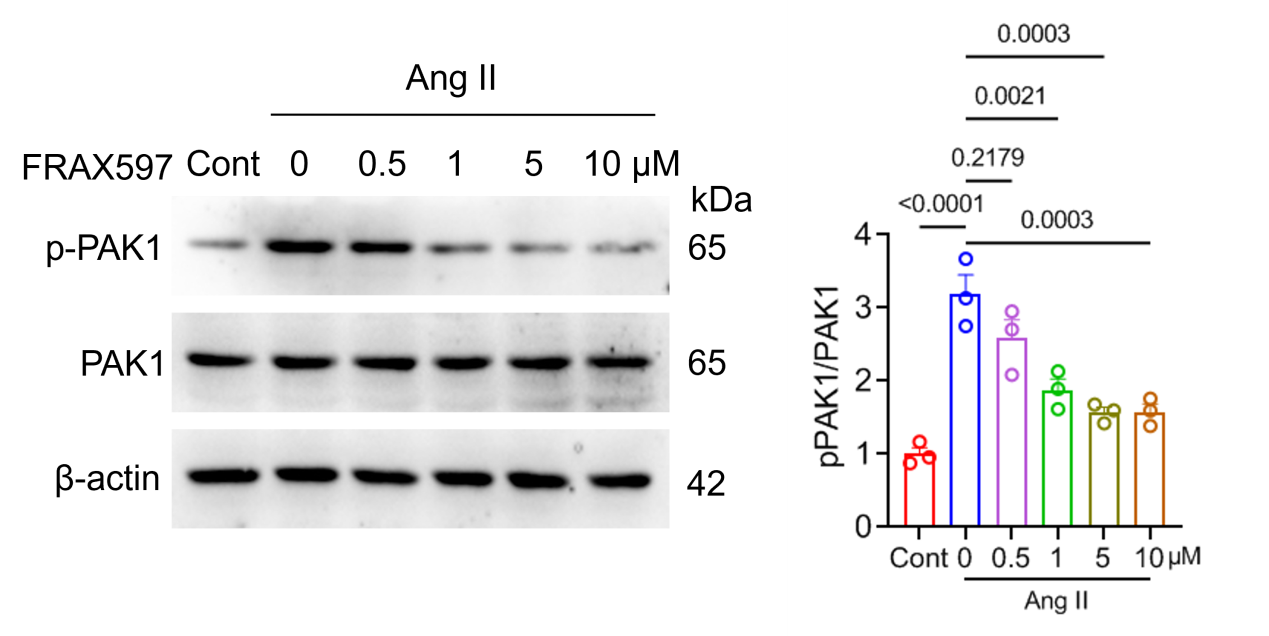


**Fig. S6. Level of p-PAK1 in VSMCs treated with various doses of FRAX597.**

VSMCs were incubated with indicated concentrations of FRAX597 for 1h, and the p-PAK1 levels were determined by Western blot. One-way ANOVA with a post hoc Tukey’s test, n=3.

**
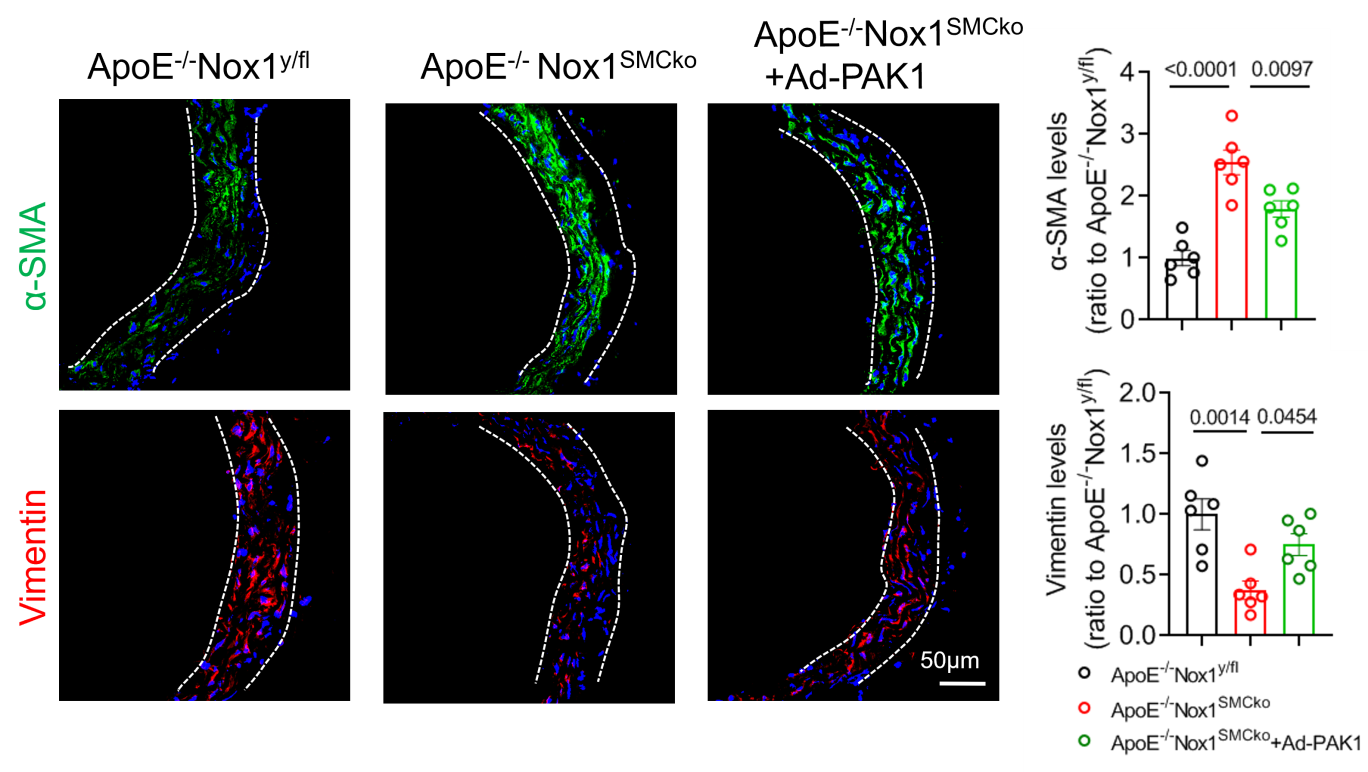
Fig. S7. Overexpression of PAK1 inhibits abdominal aortic VSMC phenotypic switching in mice of SMC-specific Nox1 deletion.**

The mice of ApoE^-/-^Nox1^y/fl^, ApoE^-/-^Nox1^SMCko^ and ApoE^-/-^Nox1^SMCko^ +Ad-PAK1 were Ang II-infused for 28 days. Cryo-sections from abdominal aortas were stained with anti-vimentin (red), anti-α-SMA (green) antibodies. Nuclei were counterstained with DAPI (blue). One-way ANOVA with a post hoc Tukey’s test, n=6.

**
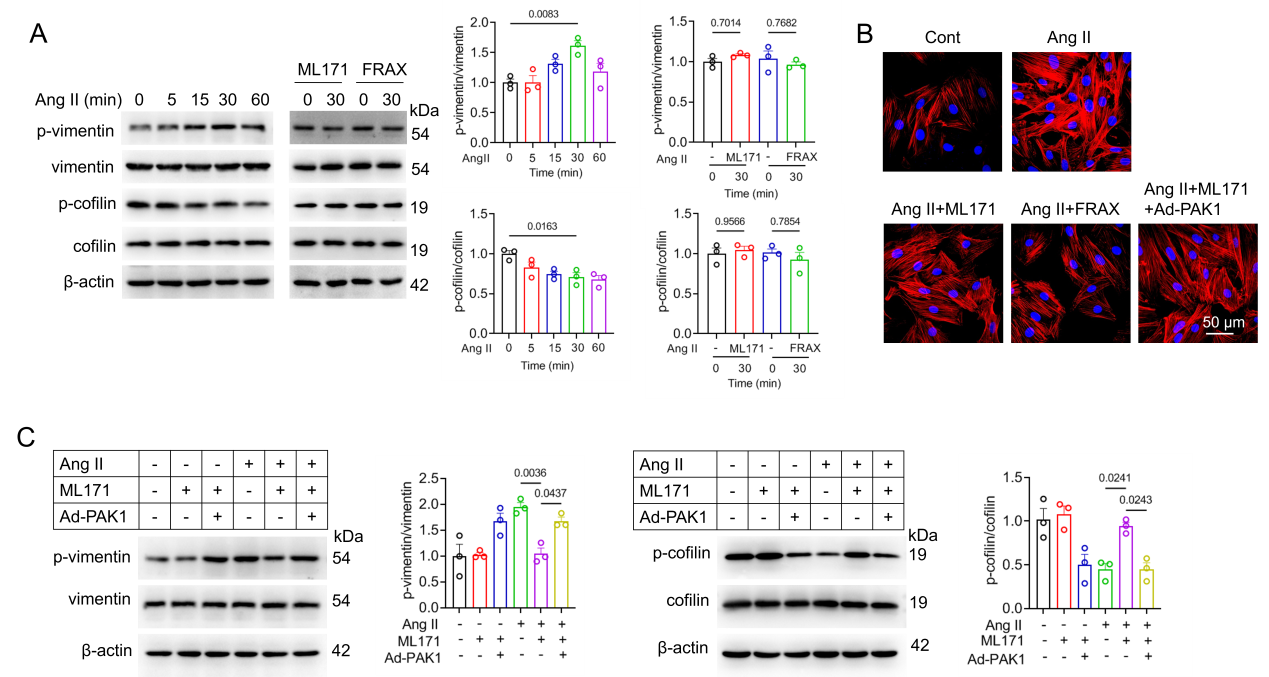
**

**Fig. S8. Nox1/PAK1 promotes cytoskeleton remodelling in Ang II-stimulated VSMCs.**

(A) Western blot analysis of p-vimentin and p-cofilin in VSMCs with indicated treatments. One-way ANOVA with a post hoc Tukey’s test (left), Student's *t*-test (right), n=3. (B) Filamentous actin (F-actin) was stained by phalloidin (red) in VSMCs treated with Ang II, Ang II+ML171 (10 μM), Ang II+FRAX (FRAX597, 1 μM) or Ang II+ML171+ Ad-PAK1. (C) VSMCs were treated as indicated for 30 min. p-vimentin and p-cofilin were determined by Western blot. Student's *t*-test, n=3.

**
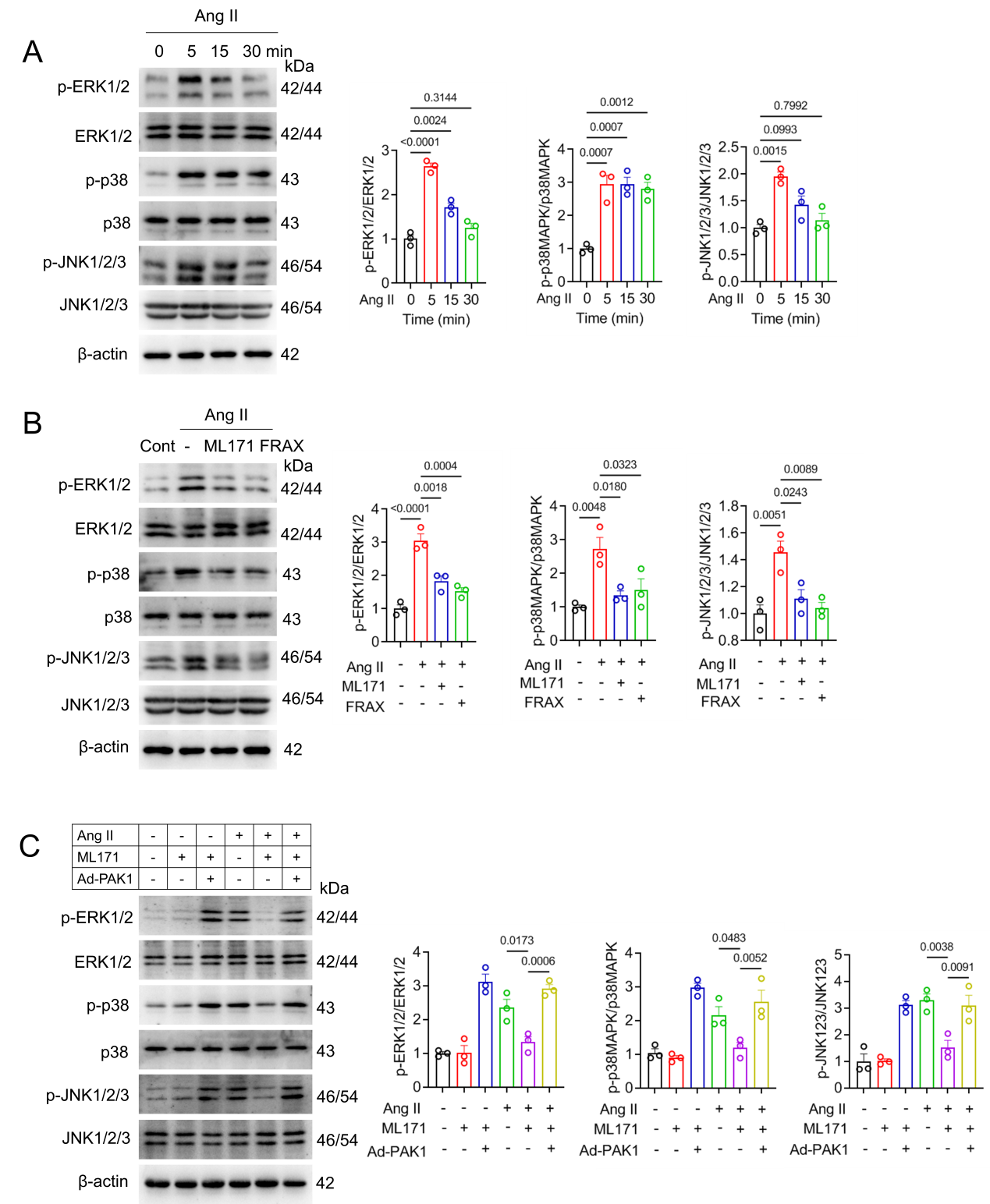
**

**Fig. S9. MAPK activity in AngII-induced VSMCs.**

(A) The VSMCs were incubated with Ang II for the indicated time periods. The p-p38, p-JNK1/2/3 and p-ERK1/2 were detected by Western blot. (B) Western blot assay for the MAPK activities including p-ERK1/2, p-p38 and p-JNK1/2/3 in VSMCs treated with Ang II, Ang II+ML171 (10 μM), or Ang II+FRAX (FRAX597, 1 μM) for 10 min. (C) Levels of p-ERK1/2, p-p38 and p-JNK1/2/3 were determined by Western blot in VSMCs treated as indicated for 10 min. One-way ANOVA with a post hoc Tukey’s test, n=3.

**
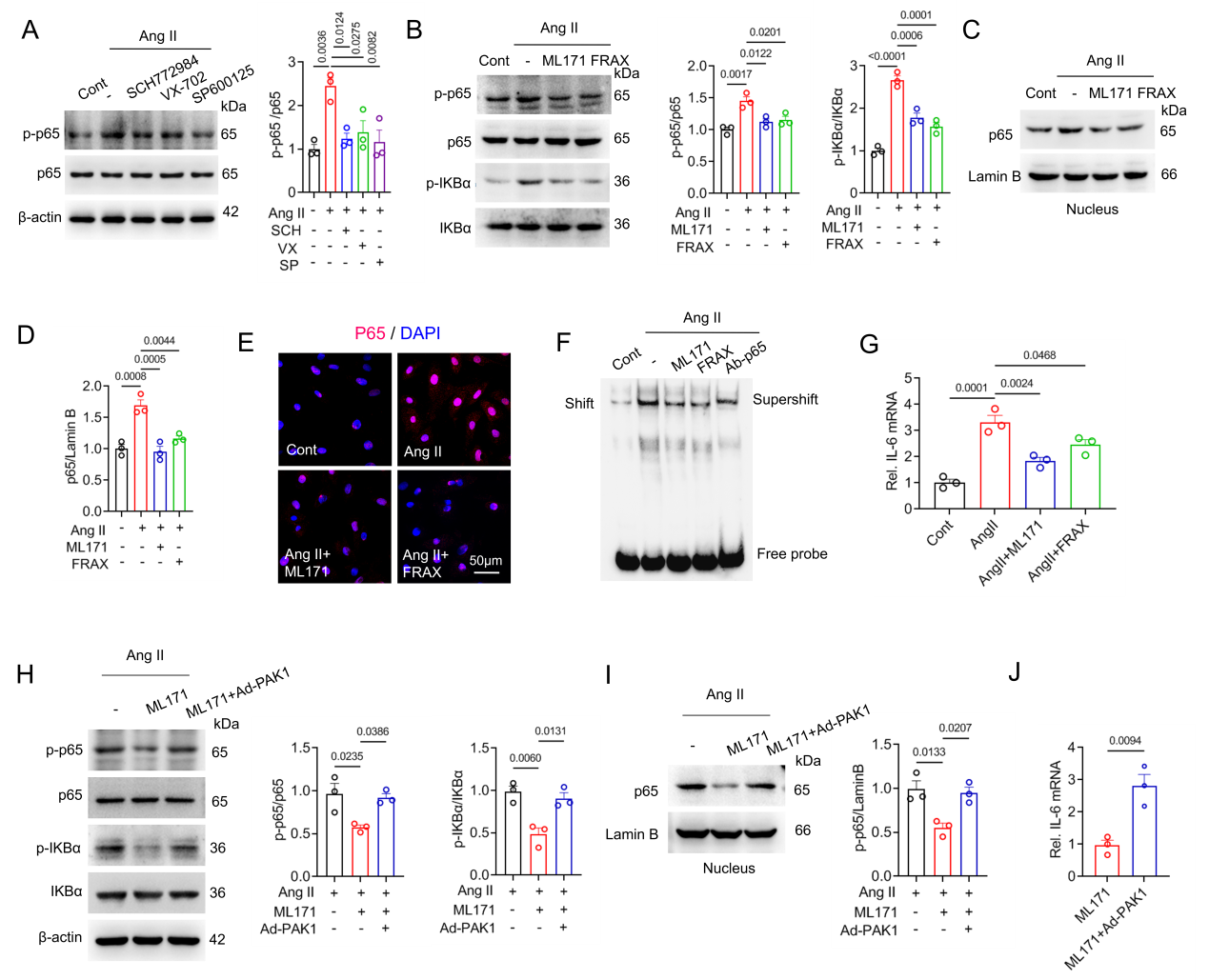
**

**Fig. S10. NFκB transcriptional activity regulated by MAPKs and Nox1/PAK1 in Ang II-induced VSMCs.**

(A) Ang II-induced VSMCs were incubated with inhibitors of ERK1/2 (SCH772894), p38 (VX-702) and JNK1/2/3 (SP600125) for 30 min. p-p65 level were detected by Western blot. (B) The p-p65 and p-IKBα levels in VSMCs treated with Ang II, Ang II+ML171, or Ang II+FRAX (FRAX597) for 30 min. (C,D) Nucleus were extracted from cells treated as the indicated, and the level of nuclear p65 was measured by immunoblotting. (E) The p65 nuclear translocation determined by immunofluorescence  staining. p65 (red), DAPI (blue). (F) EMSA for the binding of p65 with its target consensus oligonucleotides (5' -agttgaggggactttcccaggc-3') provided by Beyotime company. (G) IL-6 mRNA detected by RT-qPCR in VSMCs with indicated treatments. (H,I) VSMCs were treated as indicated for 30 min. Total p-65 and nuclear p65 were determined by Western blot. (J) Relative IL-6 mRNA levels in VSMCs treated with ML171 or ML171+Ad-PAK1 for 24 h. All the data were analyzed by one-way ANOVA with a post hoc Tukey’s test except J, n=3. Student's *t*-test in J, n=3.

**
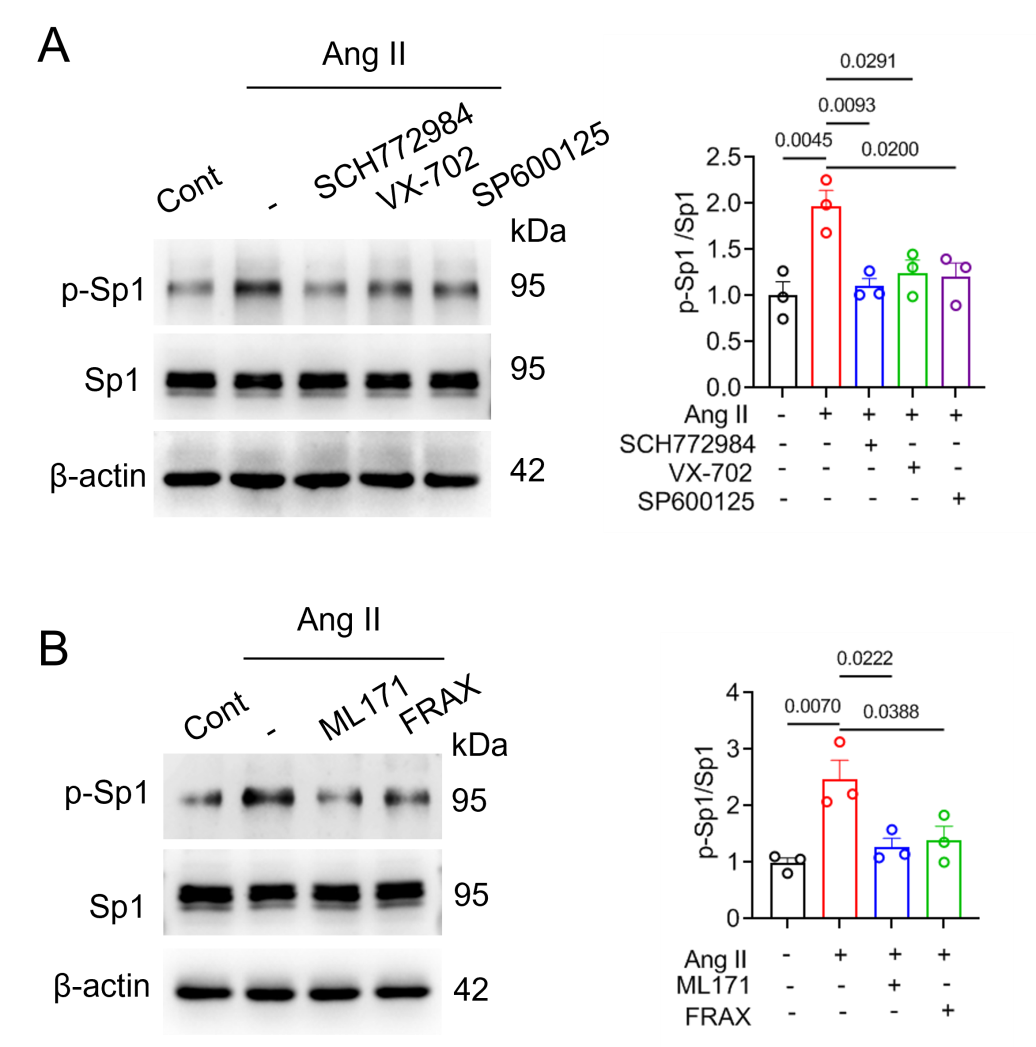
**

**Fig. S11. Sp1 activity regulated by MAPKs and Nox1/PAK1 in Ang II-induced VSMCs.**

(A) VSMCs were incubated with Ang II with or without inhibitors of ERK1/2 (SCH772894), p38 (VX-702) or JNK1/2/3 (SP600125) for 30 min. p-Sp1 level were detected by Western blot (B) The p-p65 level in VSMCs treated with Ang II, AngII+ML171, or Ang II+FRAX (FRAX597) for 30 min. The data were analyzed by one-way ANOVA with a post hoc Tukey’s test, n=3.
